# Evaluation of Exhaled Carbon Monoxide Levels in Individuals Exposed to Passive Tobacco Smoke in Indoor and Outdoor Environments: How Far Can We Getaway Under the Same Roof?

**DOI:** 10.7759/cureus.45026

**Published:** 2023-09-11

**Authors:** Dursun E Afşin, Erkut Gül, Bugra Kerget

**Affiliations:** 1 Pulmonology, University of Health Sciences, Erzurum Regional Training and Research Hospital, Erzurum, TUR; 2 Faculty of Medicine, Ataturk University, Erzurum, TUR; 3 Pulmonology, Faculty of Medicine, Ataturk University, Erzurum, TUR

**Keywords:** non-smoking, passive smoking, exhaled carbon monoxide, smoking habits, smoking tobacco

## Abstract

Introduction: Besides direct exposure, indirect contact with tobacco smoke significantly contributes to numerous health issues. Unfortunately, people are unaware that the precautions taken in closed environments are inadequate to deal with this issue. We conducted this study to assess carbon monoxide (CO) levels of people exposed to tobacco smoke indoors and outdoors.

Methods: Our study between May and June 2023 included individuals exposed to tobacco smoke indoors (n=100) and outdoors (n=100). Our control group included 100 people who had never been exposed to healthy tobacco smoke and agreed to participate in our research. The amount of CO exhaled was measured by observing how long people were in contact with tobacco smoke and how close they were to it. Questionnaires were asked of the study participants about the harms and awareness of tobacco smoke exposure.

Results: Exhaled CO levels were 1.46 ± 0.1 ppm in people exposed to tobacco smoke indoors, 1.1± 0.03 ppm in people exposed to smoke outside, and 1.1± 0.02 ppm in the control group. The statistical analysis revealed that individuals exposed to tobacco smoke in the indoor environment had significantly higher exhaled CO levels than those in the outdoor and the control groups (p=0.006). In the correlation analysis of time and distance in the indoor environment with the exhaled CO level, there was no statistically significant difference between time and space (r= -0.168, p=0.09, r=0.09, p=0.37, respectively). While less than half of both groups were aware of second-hand tobacco smoke, individuals exposed to tobacco smoke in the outdoor environment were more familiar (p<0.001).

Conclusion: Despite the precautions, indoor tobacco smoke exposure is severe because of second- and third-hand smoke. Raising individual awareness and enhancing the steps should be our top concern to prevent future health problems.

## Introduction

Smoking is an addiction that leads to multisystemic dysfunction, particularly respiratory disorders [1,2]. Despite being aware of the issue, many smokers continue to practice the habit. In Turkey, using cigarettes and tobacco products in enclosed spaces has been banned since July 2009 to tackle the problem of addiction [3]. Furthermore, higher tobacco product tariffs and the creation of smoking cessation polyclinics for people who want to quit smoking have proven helpful in reducing tobacco consumption.

Measuring exhaled carbon monoxide (CO) levels in patients who visit the smoking cessation outpatient clinic to explain the potential consequences of smoking plays a pivotal role in regulating both monitoring and demonstrating the potential harms of tobacco to persons [4]. Exhaled CO level measurement is a simple, non-invasive approach [5]. However, poor air quality and acute and chronic respiratory disorders can impact this assessment [6].

New studies have uncovered the harmful consequences of both smoking and second-hand smoke [7,8]. Recently, many areas of our country, notably in our city, have seen violations of the laws prohibiting the use of tobacco products in enclosed spaces. Smoking areas that are isolated from nonsmoking regions by a door are not in compliance with indoor environment regulations. Unfortunately, many individuals are unaware of the dangers of tobacco products and mistakenly believe they are not exposed to them.

Due to a recent increase in the areas exposed to passive cigarette and tobacco smoke, we highlight the importance of increasing social awareness in our study. The objective of this study is to evaluate the exhaled CO level in individuals who are healthy and have been exposed to passive smoke and those who have never been exposed to smoke while in an open environment. Furthermore, our study will be the first in the literature to raise awareness about our country's newly lifted sanctions.

## Materials and methods

Study design

Individuals sitting in cafés and restaurants in Erzurum between May 2023 and June 2023, where the smoking area is isolated from areas where smoking and other tobacco products are prohibited by at least 20 meters, were included. While choosing the individuals in the study, people sitting in the same area between 16.00 and 19.00 in the determined indoor environments were randomly selected. Our main purpose in selecting individuals exposed to tobacco smoke outdoors was that the weather was the same (wind speed, air temperature, atmospheric pressure). For this reason, the individuals included in the study were selected from volunteers living in the same area between 16.00 and 19.00 on the same day. Our other group consisted of healthy people exposed to passive smoke outdoors. Individuals provided verbal and written consent before being included in the study. The time spent sitting in an environment with 200 healthy people exposed to passive smoke in a smoking and tobacco-free section or in an outdoor setting and the distance between them and the area where cigarettes and tobacco products are smoked were recorded. Criteria for selecting the individuals were as follows: age, gender, job status, passive smoking levels, and acute and chronic diseases. The study covered people who did not match the following exclusion criteria. One hundred people who agreed to participate in our study and met our exclusion criteria were determined as the control group. The study was designed and conducted according to the ethical principles outlined in the Declaration of Helsinki, and Ataturk University ethics committee approval was obtained (B.30.2.ATA.0.01.00/1).

Exclusion criteria

Patients with chronic or clinically significant infectious or inflammatory conditions in the last month, current smoking, uncontrolled asthma, chronic obstructive pulmonary disease, malignancy, invasive surgery in the previous month, uncontrolled hypertension, high fasting blood glucose, or newly developed cerebrovascular disease, kidney disease, and coronary artery disease were excluded.

Definition of survey questions

Passive smoking was defined as never smoking or quitting for at least 10 years before the interview date and not currently smoking a pipe, cigar, or tobacco. Before the survey questions were asked, the individuals were given the following information on second and third-hand cigarette smoke:

Second-Hand Smoke

Second-hand cigarette exposure is defined as inhaling the primary stream smoke that the smoker emits into the surroundings and the side stream smoke that comes out of the burning end of the cigarette.

Third-Hand Smoke

Third-hand cigarette exposure is caused by inhalation or cutaneous absorption of cigarette smoke components settling on surfaces and objects in the smoking area.

Following the information given, the questions were answered yes/no.

Exhaled CO measurement

Exhaled CO and COHb% levels were measured using a Pico™ Smokerlyzer® (Bedfont Scientific LTD, Kent, UK). The patients were instructed to inhale deeply, hold for 15 seconds, and slowly exhale until their lungs were empty. The device was calibrated before the test began, and the participants who performed the trial were given a disposable mouthpiece.

Statistical analysis

Statistical analysis was performed using IBM SPSS Statistics for Windows, Version 22 (Released 2013; IBM Corp., Armonk, New York, United States). Between-group comparisons were performed using Pearson’s chi-square test for parametric data and the Mann-Whitney U test for non-normally distributed numerical data. Demographic data and laboratory parameters were compared between the groups using the Kruskal-Wallis test. Variables that showed significant differences in the Kruskal-Wallis test were compared among groups using an independent samples t-test. Spearman correlation analysis evaluated the relationship between laboratory parameters and radiological scores. A p-value less than 0.05 was considered statistically significant.

## Results

The average age of the participants in the study was 25.6±6.8. While the mean age of people exposed to tobacco smoke indoors was 26.1±3.3, it was 25.8± 4.1 in people exposed to it outdoors. The study's control group included people who ranged in age from 25.5 ± 5.3 years. The statistical analysis of the mean age of the subjects revealed no significant differences (p=0.89).

It was discovered that 52% of those in the indoor environment, 51% in the outdoor environment, and 53% in the control group were male. It was also discovered that there was no statistically significant difference between the genders (p=0.9).

Table 1 shows the awareness levels of the people in our study who were exposed to passive tobacco smoke about the dangers of tobacco smoke in their surroundings and the tobacco use regulations. According to the analysis, individuals exposed to tobacco smoke indoors were more hazardous than outdoors (p<0.001). While less than half of both groups had information on second-hand smoke, persons accustomed to tobacco smoke in the open environment were more aware (p<0.001). Sadly, it was discovered that neither group had enough information about third-hand smoke exposure.

**Table 1:** Demonstration of the degrees of awareness of people exposed to tobacco products both indoors and outdoors The patients who answered yes to the questions asked were stated as a percentage (%). p-value <0.05 was considered statistically significant.

	Those exposed to indoor tobacco smoke (n=100) (Yes = %)	Those exposed to outdoor tobacco smoke (n=100) (Yes = %)	p
Do you believe exposure to tobacco smoke in your environment is dangerous?	76	20	<0.001
Are you aware that it is illegal to consume tobacco products indoors?	98	100	0.9
Do you know about the dangers of passive tobacco smoke exposure?	63	65	0.9
Do you know anything about second-hand smoke?	8	34	<0.001
Do you know anything about third-hand smoke?	1	-	N/A
Are you aware of the risks of second-hand cigarette smoke in public places?	2	1	N/A
Is it healthier to be exposed to cigarette smoke outside than inside?	95	100	0.8

**Figure 1:**
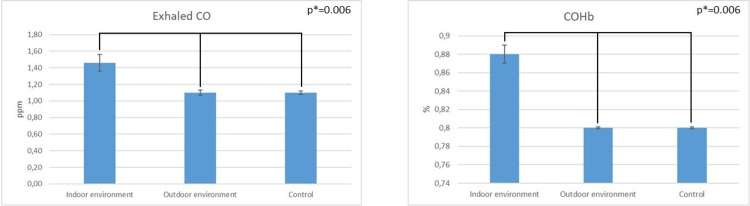
Comparison of exhaled CO levels of individuals exposed to tobacco smoke in indoor and outdoor environments and the control group p-value <0.05 was considered statistically significant.

Figure 1 depicts the measurement of exhaled CO and CoHb% levels, forming our investigation's basis. What stands out in the table is that the exhaled CO level in persons exposed to tobacco smoke in an indoor setting was 1.46 ± 0.1 ppm, 1.1±0.03 ppm in individuals exposed to tobacco smoke in an open environment, and 1.1±0.02 ppm in individuals in the control group. The statistical analysis of the groups revealed that people who consumed tobacco smoke in the indoor environment had significantly greater exhaled CO levels than those in the outdoors and the control group (p=0.006). The COHb% values for individuals exposed to indoor tobacco smoke were 0.88 ± 0.01 ppm, while those exposed to outdoor smoke were 0.8 ± 0.001 ppm. The COHb% value was also 0.8 ± 0.001 ppm for those in the control group. Statistical analysis found that individuals exposed to tobacco smoke indoors had significantly higher COHb% levels compared to both groups (p=0.006).

The relationship between exhaled CO levels and the time and distance of individuals exposed to tobacco smoke between specified time zones in an indoor environment was evaluated. In the correlation analysis of time and distance in the indoor environment with the exhaled CO level, there was no statistically significant difference between time and distance (r= -0.168, p=0.09, r=0.09, p=0.37, respectively).

## Discussion

Many studies have been conducted on the harms of exposure to tobacco smoke in open and closed environments. In the meta-analysis in which the studies were compiled, it was concluded that exposure to tobacco smoke in indoor environments would cause more particles to be inhaled [9]. However, it is also thought that atmospheric conditions may have significant effects on the amount of particles in outdoor tobacco smoke exposure. The main purpose of these studies is to increase public awareness about the harms of tobacco smoke. Unfortunately, it is observed in our study that this awareness is not sufficient. In addition, higher exhaled CO levels were observed in individuals exposed to tobacco smoke in a closed environment, which is also consistent with the literature. Exposure to third-hand tobacco smoke has come to the fore more recently. However, there are no sufficient studies on the effect of this situation on the exhaled CO level. Our study also reveals important results about the harms of third-hand tobacco smoke exposure.

Tobacco smoke may cause diseases in numerous systems and organs, specifically the lungs. This sort of situation is known to occur in people who are both passive smokers and directly impacted by tobacco smoke. According to studies, some harmful substances are more prevalent in the side stream noticed in cigarette smoke. The most common source of comorbidities among passive smokers is sidestream smoke [10].

Exposure to second-hand cigarettes is inhaling the primary stream smoke that the smoker emits into the surroundings and the side stream smoke that comes out of the burning end of the cigarette [8]. On the other end of the spectrum, third-hand cigarette exposure means exposure to objects in the smoking environment due to inhalation or cutaneous absorption of cigarette smoke components settling on surfaces [11,12]. According to a World Health Organization research study released in 2013, one-third of the global population is exposed to second-hand smoke. Most notably, women between the ages of 15-49 who are of childbearing age make up half of the household's passive smokers. This exposure is responsible for the deaths of 600,000 individuals each year, approximately one-third of whom are children [13,14].

Passive smoke exposure has been associated with various ailments, particularly lung and cardiovascular disease. According to the meta-analyses, it increases by 24% in those who live in the same house as a smoker compared to individuals who live in a residence where no one has ever smoked [14]. Passive smoking has been proven to cause recurrent lower and upper respiratory tract infections, especially in children [15,16]. The situation was unrelated to the mother's smoking while pregnant [17]. The use of tobacco products indoors was outlawed in Turkey in January 2008 due to this understanding spreading throughout many nations, including ours [18]. While this rule was strictly enforced due to heightened precautions when the law was issued, the economic devastation caused by the COVID-19 outbreak in indoor cafes has relaxed these rules. Even though people who do not want to be directly exposed to tobacco smoke prefer to live where tobacco products are not consumed, we discovered that they are frequently unaware of the risks of passive exposure.

According to our findings, individuals exposed to tobacco smoke indoors and outdoors are sufficiently mindful of tobacco use indoors. Nevertheless, we discovered that persons in a closed environment believe they are in danger because regulations are not followed, notwithstanding the current legal consequences. We found that people exposed to indoor tobacco smoke had less knowledge of second-hand smoke than those exposed outdoors. Likewise, we noticed that both groups needed an appropriate understanding of third-hand smoke. In terms of inhaled CO levels, we discovered that people exposed to tobacco smoke indoors had higher levels than both the outdoor and control groups. The correlation analysis indicated that this situation was independent of location and duration in people exposed to indoor cigarette smoke. When the results of individuals exposed to tobacco smoke indoors and outdoors are evaluated together, it may be concluded that exposure to third-hand smoke may cause worse outcomes. The absence of information about the potential hazards of third-hand smoke exposure demonstrates how critical this situation is for spreading awareness.

A limitation of this study is the difficulty of standardizing items that potentially affect third-hand smoke exposure in locations where tobacco smoke was present. Nevertheless, exposure time and distance to indoor tobacco smoke do not correlate with exhaled CO level, demonstrating the hazard of third-hand smoke exposure even in a non-standardized scenario.

## Conclusions

As a result, in our study, it was seen that individuals exposed to tobacco smoke outdoors had more knowledge about the harms of exposure to tobacco smoke. This may have caused individuals to be outdoors with tobacco smokers. However, the fact that both groups do not have enough information about third-hand smoke shows that informing them about the great danger is insufficient. Individuals may think that the more they stay away from indoor tobacco smoking areas, the more protected they will be. However, our study data show that this is not as expected. However, exposure to tobacco smoke outdoors seems safer than indoors. Raising public awareness about exposure to tobacco smoke should be one of our most essential duties in terms of public health.
